# Concomitant Venous Disease in Patients with Advanced Peripheral Arterial Disease: A Patient- and Limb-Level Analysis

**DOI:** 10.3390/life16020312

**Published:** 2026-02-11

**Authors:** Daniela Marinescu, Laurențiu Augustus Barbu, Tiberiu Stefăniță Țenea Cojan, Ștefan Pătrascu, Marius Bică, Răzvan Alexandru Marinescu, Sarmis Marian Săndulescu, Valeriu Șurlin, Ana-Maria Ciurea

**Affiliations:** 1 Department of Surgery, Emergency County Hospital, University of Medicine and Pharmacy of Craiova, 2 Petru Rares Street, 200349 Craiova, Romania; dtmarinescu@yahoo.com (D.M.); stefan.patrascu@umfcv.ro (Ș.P.); marius.bica@umfcv.ro (M.B.); ssarmis@yahoo.com (S.M.S.); 2 Department of Surgery, Railway Clinical Hospital Craiova, University of Medicine and Pharmacy of Craiova, 2 Petru Rares Street, 200349 Craiova, Romania; tiberiu.tenea@umfcv.ro; 3 Department of Plastic Surgery, University of Medicine and Pharmacy of Craiova, 200349 Craiova, Romania; razvanalexandrumarinescu98@gmail.com; 4 Academy of Romanian Scientist, 3 Ilfov Street, 050044 Bucharest, Romania; 5 Department of Oncology, Emergency County Hospital, University of Medicine and Pharmacy of Craiova, 2 Petru Rares Street, 200349 Craiova, Romania; amciurea14@gmail.com

**Keywords:** peripheral arterial disease, chronic limb-threatening ischemia, venous disease, venous dysfunction, lower-limb edema, arterio-venous interaction, limb-threatening ischemia, amputation risk, vascular assessment

## Abstract

**Background:** Advanced stages of peripheral arterial disease, particularly chronic limb-threatening ischemia, are characterized by unfavorable limb outcomes and a substantial risk of major amputation. Clinical evaluation traditionally focuses on arterial obstruction; however, venous dysfunction may coexist and contribute to local limb pathophysiology in advanced PAD, remaining insufficiently recognized in routine practice. **Methods:** We performed a retrospective cohort analysis of consecutive patients with advanced peripheral arterial disease managed at the First Surgical Clinic of the Emergency County Clinical Hospital of Craiova over a five-year period (January 2020 to December 2024). Venous disease was defined using a clinically oriented composite definition incorporating imaging-confirmed venous pathology, prior deep venous thrombosis, and persistent lower-limb edema attributable to venous dysfunction. Arterial disease severity was assessed using multimodal imaging. Analyses were performed at both patient and limb levels to evaluate associations between venous disease, arterial severity markers, and clinical outcomes. **Results:** Among 241 patients (482 limbs), concomitant venous disease was identified in 68.9% at the patient level and was predominantly unilateral. At the limb level, venous disease was significantly associated with markers of severe arterial involvement, including inflow disease, higher segment occlusion scores, impaired tibial runoff, and absence of a patent pedal arch. Despite greater arterial severity, patients with venous disease exhibited a lower unadjusted rate of major amputation compared with those without venous involvement. **Conclusions:** Concomitant venous disease is highly prevalent in patients with advanced PAD and is closely linked to arterial disease severity. These findings suggest that venous dysfunction represents an integral component of advanced limb-threatening ischemia rather than an isolated comorbidity. Incorporating clinically oriented venous assessment may improve understanding of limb pathophysiology and support a more integrated arterio-venous approach to advanced PAD management.

## 1. Introduction

Peripheral arterial disease (PAD), particularly in its advanced stage as chronic limb-threatening ischemia (CLTI), is associated with an unfavorable clinical trajectory marked by high risks of major limb loss and death. Although current guidelines prioritize arterial revascularization, a considerable subset of patients cannot undergo endovascular or surgical treatment because of advanced frailty, substantial comorbidity burden, or complex arterial anatomy. Contemporary evidence indicates that clinical outcomes in these patients remain poor, with persistently high rates of major amputation and mortality at one year, and limited long-term amputation-free survival among individuals deemed unsuitable for revascularization [1,2].

Beyond ischemic manifestations confined to the limb, patients with advanced PAD constitute a clinically vulnerable population characterized by a substantial burden of systemic comorbidities, including cardiovascular disease, diabetes mellitus, chronic kidney dysfunction, and frailty. Large registry-based studies have demonstrated that this patient group is progressively older and medically more complex, exhibiting multimorbidity profiles that strongly shape therapeutic decision-making and influence clinical outcomes [3,4].

While ischemic ulceration and the anatomical extent of atherosclerotic disease remain central to the clinical assessment of CLTI, growing evidence indicates that limb prognosis cannot be explained by arterial obstruction alone. Factors such as distal tissue perfusion, microcirculatory impairment, and local wound-related conditions play a pivotal role in healing trajectories and limb preservation, even following technically successful proximal revascularization [5,6]. Together, these findings highlight the multifactorial pathophysiology of limb-threatening ischemia and expose the limitations of a purely arterial-focused evaluative framework.

Within this framework, concomitant venous disease emerges as a potentially important yet insufficiently investigated component of vascular pathology in advanced PAD. Venous hypertension, lower-limb edema, and impaired venous outflow may further disrupt tissue oxygen delivery, aggravate microcirculatory dysfunction, and impede wound healing, particularly in limbs already compromised by severe arterial insufficiency [7,8]. Imaging-based investigations have documented the presence of chronic venous insufficiency in a considerable proportion of patients with peripheral arterial disease and have linked venous abnormalities to more advanced stages of arterial disease. Notably, venous pathology often remains inadequately recorded in routine clinical documentation, suggesting that combined arterio-venous disease is frequently underrecognized [9,10].

Moreover, accumulating evidence indicates that major limb loss in PAD reflects the interplay between chronic ischemia, superimposed infection, impaired tissue repair, and patient-related vulnerability, rather than arterial insufficiency in isolation [11]. In this multifactorial context, concomitant venous disease may function as an additional determinant of the local limb environment; however, its true prevalence and clinical relevance remain incompletely defined.

Accordingly, this study sought to quantify the burden of concomitant venous disease in patients with advanced peripheral arterial disease and to explore its relationship with arterial anatomical severity and clinically relevant outcomes, applying both patient-level and limb-level analytical frameworks.

## 2. Materials and Methods

### 2.1. Study Design and Population

This retrospective observational cohort study was conducted at the First Surgical Clinic of the Emergency County Clinical Hospital of Craiova, Romania. Consecutive patients with advanced peripheral arterial disease (PAD) who were evaluated and treated between 1 January 2020 and 31 December 2024 were screened for inclusion.

Eligible patients were adults (≥18 years) diagnosed with advanced PAD requiring inpatient vascular evaluation and/or intervention. Both patient-level and limb-level analyses were performed. When applicable, each lower limb was considered an independent analytical unit for limb-level assessments.

Baseline demographic data and major comorbidities were extracted from the medical records. Hyperlipidemia was recorded only when explicitly documented as a prior diagnosis in the medical records. Lipid abnormalities identified solely on laboratory testing or the use of lipid-lowering therapy in the absence of a documented diagnosis were not systematically captured, reflecting the retrospective nature of the study.

### 2.2. Inclusion and Exclusion Criteria

Inclusion criteria were:

- Diagnosis of advanced PAD, including chronic limb-threatening ischemia (CLTI);
- Availability of complete clinical, imaging, and procedural data;
- Documented arterial imaging, allowing anatomical assessment.

Exclusion criteria were:

- Acute limb ischemia;
- Prior major amputation above the ankle before index admission;
- Active systemic infection unrelated to limb pathology at admission;
- Incomplete clinical or imaging records.

### 2.3. Assessment of Arterial Disease

Arterial disease severity was assessed using multimodal imaging according to institutional protocols, including:

- Arterial duplex Doppler ultrasonography;
- Computed tomography angiography (CTA);
- Digital subtraction angiography.

Arterial involvement was characterized by:

- Presence of inflow, femoropopliteal, and infrapopliteal disease;
- Segmental occlusion burden;
- Tibial runoff status;
- Pedal arch patency.

Arterial disease severity was analyzed at both the patient and limb levels.

### 2.4. Definition and Assessment of Venous Disease

Venous disease was defined using a clinically oriented composite definition, designed to reflect real-world vascular practice rather than restrictive imaging-only criteria.

Venous disease was considered present if at least one of the following criteria was documented:

- Imaging-confirmed chronic venous insufficiency on duplex Doppler ultrasonography;
- Documented history of deep venous thrombosis;
- Persistent lower-limb edema attributable to venous dysfunction, in the absence of acute cardiac, renal, hepatic, infectious, or inflammatory causes.

Lower-limb edema was primarily assessed clinically and defined as objectively documented swelling on physical examination. Venous duplex Doppler ultrasonography was used as a complementary tool to support the presence of venous dysfunction; however, Doppler confirmation was not mandatory. Among patients classified as having venous disease, 69/166 (41.6%) had imaging-confirmed venous pathology on duplex Doppler ultrasonography. In the remaining cases, venous disease was identified based on documented prior deep venous thrombosis or clinically significant persistent edema after exclusion of non-venous causes, reflecting real-world clinical assessment in patients with advanced PAD. This approach allowed identification of clinically meaningful functional venous impairment.

The explicit inclusion of edema aimed to capture venous dysfunction that may substantially influence limb pathophysiology and outcomes but is frequently excluded from studies relying exclusively on imaging-based definitions.

Venous disease assessment was performed at both the patient and limb levels.

### 2.5. Outcomes

The study outcomes were predefined to ensure clarity and methodological focus, particularly given the retrospective design and the limited sample size.

The primary outcome of the study was Major Adverse Limb Events (MALE), defined at the patient level as the occurrence of major amputation (above the ankle) or limb reintervention during follow-up.

The secondary outcomes included:

1. Major amputation, analyzed as an individual endpoint;
2. Limb salvage;
3. All-cause mortality during follow-up;
4. Associations between concomitant venous disease and markers of arterial disease severity.

Variables describing arterial anatomy and ischemic burden—such as inflow disease, segment occlusion score, tibial runoff, and pedal arch patency—were analyzed as descriptive or exploratory parameters and were not considered clinical outcome measures.

The selection of a single primary outcome was intended to reduce multiplicity and avoid overinterpretation of results, in accordance with commonly used limb-related outcome frameworks and the sample size of the study.

### 2.6. Statistical Analysis

Continuous variables are presented as mean ± standard deviation or median (interquartile range), as appropriate. Normality of continuous variables was assessed using the Shapiro–Wilk test. Parametric or non-parametric statistical tests were selected accordingly, based on distribution characteristics. Categorical variables are expressed as counts and percentages.

Comparisons between groups were performed using Student’s *t*-test or Mann–Whitney *U* test for continuous variables and the χ^2^ test or Fisher’s exact test for categorical variables.

Multivariable logistic regression analyses were used to identify independent predictors of venous disease and limb-related outcomes. Variables entered into multivariable regression models were selected based on clinical relevance and prior evidence, as well as univariable associations with the outcome of interest. Given the advanced and highly unbalanced arterial disease distribution, potential collinearity among arterial severity markers was considered, and regression analyses were interpreted as exploratory. Results are reported as odds ratios (ORs) with 95% confidence intervals (CIs). Statistical significance was defined as a two-sided *p* value < 0.05.

All statistical analyses were performed using SPSS software, version 25.0 (IBM Corp., Armonk, NY, USA).

Regression analyses exploring predictors of venous disease were performed as secondary, exploratory analyses to contextualize the distribution of venous involvement, whereas limb-related outcomes were analyzed in relation to venous disease status. Given the advanced and highly unbalanced arterial disease distribution, multivariable regression analyses were considered exploratory, and results were interpreted in the context of potential collinearity among arterial severity markers.

### 2.7. Ethical Considerations

The study was conducted in accordance with the Declaration of Helsinki and was approved by the Ethics Committee of the Emergency County Clinical Hospital of Craiova (approval number 55514/18 November 2025).

Written informed consent for the use of anonymized medical data was obtained from all patients at the time of hospital admission.

## 3. Result

As shown in Table 1, baseline demographic characteristics and major comorbidities were comparable between patients with and without concomitant venous disease. Age and sex distribution did not differ significantly between groups. Similarly, the prevalence of hypertension, diabetes mellitus, hyperlipidemia, smoking history, congestive heart failure, prior stroke, and chronic kidney disease requiring hemodialysis was high and evenly distributed across both groups (all *p* > 0.05).

These findings indicate that the presence of venous disease was not associated with differences in baseline clinical characteristics, suggesting that venous involvement in this cohort is unlikely to be driven by demographic factors or comorbidity burden and may instead reflect disease-specific vascular characteristics explored in subsequent analyses.

Venous disease was present in 68.9% of patients (166/241). Among these, unilateral venous disease was observed in 55.6% of patients (134/241), while bilateral venous disease was identified in 13.3% (32/241) (Table 2). At the limb level, venous disease was more frequent in the left limb (49.0%) compared with the right limb (20.7%). These findings indicate that concomitant venous disease is common in patients with peripheral arterial disease, with a predominance of unilateral involvement.

Limb-level analysis demonstrated a severe and extensive pattern of peripheral arterial disease (Table 3). Inflow disease was present in 93.7% of limbs, femoropopliteal disease in 65.6%, and outflow disease in 98.3%, with multilevel involvement identified in 96.6% of cases. The median segment occlusion score was 4.0 [IQR 1.0–4.0], indicating advanced obstructive disease. Distal perfusion was markedly compromised, with a median tibial runoff count of 1.0 [IQR 0.0–2.0] and a patent pedal arch present in only 33.6% of limbs.

Venous disease was associated with increased PAD severity at the limb level. Its prevalence was higher in the presence of inflow disease and in limbs without a patent pedal arch (Table 4). A clear gradient was observed, with venous disease increasing across higher segment occlusion scores and worsening tibial runoff, reaching the highest prevalence in limbs with severe distal ischemia.

Treatment strategies were largely comparable between patients with and without venous disease, with no significant differences in the use of endovascular therapy, angioplasty, thromboendarterectomy, or bypass surgery (Table 5). However, patients with concomitant venous disease showed a lower unadjusted rate of amputation compared with those without venous involvement (22.3% vs. 44.0%, *p* = 0.001), while rates of documented occlusion and mortality during follow-up were similar between groups.

In multivariable logistic regression analysis, venous disease was independently associated with specific arterial characteristics (Table 6). The presence of a patent pedal arch was significantly associated with higher odds of venous disease (OR 3.00, 95% CI 1.64–5.52, *p* < 0.001), as was the presence of inflow disease (OR 94.20, 95% CI 2.55–3478.01, *p* = 0.014). Age, sex, segment occlusion score, and tibial runoff did not show independent associations with venous disease after adjustment.

## 4. Discussion

In this study, concomitant venous disease was found to be highly prevalent among patients with advanced peripheral arterial disease, affecting nearly 70% of the cohort. Venous involvement was predominantly unilateral, while bilateral disease was observed in a smaller proportion of cases, supporting the notion that venous dysfunction in advanced PAD is more often localized rather than a manifestation of systemic venous pathology. This prevalence exceeds that reported in several previous PAD series, in which concomitant venous disease has generally been documented in approximately 10–25% of patients. However, this difference is more plausibly attributable to methodological and clinical variations across studies than to an overestimation in the present analysis.

Notably, population-based and imaging-focused investigations consistently indicate that venous pathology is frequently underrecognized in patients with PAD, largely because routine vascular assessment prioritizes arterial patency and ischemic burden. Studies that exclude individuals with documented venous disease or that lack systematic venous evaluation are therefore likely to underestimate the true extent of combined arterio-venous pathology. Imaging analyses have demonstrated that a substantial proportion of PAD patients with venous insufficiency lack prior clinical documentation, particularly in advanced stages of disease [12,13]. Together, these observations reinforce our findings that venous disease constitutes a clinically meaningful yet often overlooked component of advanced PAD, especially in limb-threatening presentations.

This arterial-focused diagnostic paradigm is not confined to PAD populations. In surgical cohorts of patients with advanced pelvic malignancies previously treated with radiotherapy, comprehensive intraoperative evaluation has revealed frequent venous and perivascular abnormalities that were not evident on preoperative imaging and did not consistently correlate with overt macrovascular arterial disease [14,15,16]. These alterations—including fibrotic changes, disrupted venous outflow patterns, and microvascular remodeling—reflect extensive local vascular remodeling induced by malignancy and oncologic treatment. Such observations underscore how clinically meaningful venous and microvascular pathology may remain unrecognized when vascular assessment is centered primarily on arterial anatomy. Comparable diagnostic limitations may therefore contribute to the systematic underestimation of venous disease in patients with advanced PAD, particularly in the absence of standardized or clinically oriented venous evaluation.

Most previous investigations have relied on restrictive definitions of venous disease, predominantly limited to imaging-confirmed abnormalities detected by duplex ultrasonography, such as direct or indirect signs of chronic venous insufficiency or a documented history of deep venous thrombosis [17]. In our cohort, the proportion of patients with imaging-confirmed venous pathology is comparable to that reported in earlier PAD series, in which venous duplex evaluation is typically performed selectively based on clinical suspicion rather than applied systematically. By contrast, venous disease in the present study was defined using a clinically oriented framework that also incorporated functional manifestations, including persistent limb edema, thereby capturing clinically meaningful venous dysfunction. Although this approach is expected to yield higher prevalence estimates than imaging-only definitions, its clinical relevance is supported by imaging-based evidence. In a magnetic resonance angiography study, concomitant chronic venous insufficiency was identified in approximately one-fifth of patients with PAD, with a substantial proportion lacking prior clinical documentation; importantly, combined arterial and venous pathology was associated with more advanced PAD stages [12,18].

Second, the arterial disease characteristics of our cohort indicate an exceptionally severe PAD phenotype. Limb-level analyses revealed near-universal multilevel and outflow involvement, elevated segment occlusion scores, markedly reduced tibial runoff, and a low prevalence of patent pedal arches. This extent of arterial compromise is typical of advanced limb-threatening disease, in which chronic hypoperfusion, microcirculatory impairment, inflammatory processes, and local hemodynamic disturbances may facilitate the development and persistence of venous pathology. Accordingly, the high prevalence of venous disease observed in this study likely reflects the selection of a particularly severe vascular phenotype rather than a true discrepancy with existing literature.

Patients with advanced PAD represent a clinically heterogeneous and medically complex group, typically characterized by older age, substantial multimorbidity, and wide variability in both limb-specific and systemic disease severity [19]. Contemporary large-scale registry analyses have demonstrated that intersecting cardiovascular, metabolic, and renal comorbidities exert a major influence on vascular pathophysiology and clinical outcomes in this population [3,4,20]. Within this clinical landscape, the coexistence of venous disease may be more prevalent than previously recognized, particularly among individuals with severe distal ischemia and advanced tissue involvement.

Moreover, population-based and imaging-oriented studies consistently suggest that venous pathology is frequently underdetected in PAD cohorts, in part because routine vascular assessment remains predominantly focused on arterial patency and ischemic burden [21]. As a result, investigations that do not incorporate systematic venous evaluation are likely to underestimate the true extent of combined arterio-venous disease [22,23,24]. Consistent with these observations, the present findings underscore venous disease as a clinically meaningful yet commonly overlooked component of advanced PAD, especially in limb-threatening clinical scenarios.

At the limb level, venous disease demonstrated strong associations with established indicators of PAD severity, including inflow involvement, higher segment occlusion scores, absence of a patent pedal arch, and progressively impaired tibial runoff. These relationships align with pathophysiological evidence suggesting that severe arterial ischemia elevates interstitial pressure, disrupts endothelial integrity, and compromises venous outflow, thereby fostering a self-reinforcing arterio-venous feedback loop. Within this framework, limb outcomes in CLTI appear to be governed not by arterial obstruction in isolation, but by the interplay of macrovascular ischemia, microcirculatory impairment, infection, wound-related factors, and patient-specific vulnerability, rather than by inadequate arterial revascularization alone [25]. The present findings extend this concept by highlighting venous disease as a clinically meaningful modifier of the local limb environment in advanced PAD.

A noteworthy and seemingly counterintuitive observation was the lower unadjusted rate of major amputation among patients with concomitant venous disease, despite greater arterial severity at the limb level. This finding challenges the conventional assumption that venous pathology uniformly worsens limb prognosis [26]. One plausible interpretation is that patients with venous disease represent a distinct clinical phenotype in which venous-related manifestations, such as edema, may prompt earlier clinical attention, closer follow-up, and potentially more proactive limb salvage efforts. These hypotheses remain speculative, as key variables—including infection burden, wound characteristics, timing of presentation, and revascularization intent—were not systematically captured in this retrospective cohort. Furthermore, population-based evidence indicates that major limb loss in PAD and CLTI is driven not solely by arterial insufficiency, but by the convergence of ischemia and infection [27,28,29]. Within this multifactorial setting, venous disease—through its associations with edema, impaired wound healing, and heightened susceptibility to infection—may therefore modulate limb prognosis rather than exert a uniformly detrimental effect.

Recent population-based investigations have demonstrated that outcomes in CLTI are shaped not only by anatomical disease burden but also by clinical trajectories and the timing of therapeutic intervention [30]. Delays in revascularization have been independently linked to higher mortality, particularly among patients presenting with tissue loss, underscoring the importance of timely recognition and optimized limb management pathways [31,32,33,34]. Within this context, venous-related clinical features—such as lower-limb edema or wound exudation—may facilitate earlier presentation and referral, thereby potentially influencing limb-related outcomes in patients with concomitant venous disease.

The association between venous disease and pedal arch patency deserves specific attention. At the limb level, venous disease was more frequently observed in the absence of a patent pedal arch, consistent with more advanced distal ischemia and compromised local perfusion. Conversely, patient-level multivariable analysis identified the presence of a patent pedal arch as an independent correlate of venous disease. This apparent inconsistency likely reflects differences in analytical scale, collinearity among arterial severity metrics, and the markedly unbalanced distribution of arterial lesions in this cohort with advanced PAD. The broad confidence intervals observed in the multivariable models further warrant cautious interpretation and highlight the challenges of disentangling arterial and venous contributions in end-stage vascular disease. Collectively, these findings suggest that venous disease in advanced PAD is predominantly driven by local, limb-specific hemodynamic factors rather than representing a uniform, bilateral systemic process.

Taken together, our findings align with emerging literature describing a close and clinically relevant interaction between the arterial and venous systems in the lower limb. The present study extends these observations by demonstrating a high prevalence of venous disease in a cohort with advanced PAD and by systematically linking venous involvement to objective markers of arterial severity at both patient and limb levels. These results support the need for a more integrated arterio-venous approach in the evaluation and management of patients with advanced peripheral arterial disease.

### 4.1. Strengths and Limitations

This study provides a comprehensive assessment of concomitant venous disease in patients with advanced peripheral arterial disease using both patient- and limb-level analyses. The use of a clinically oriented composite definition of venous disease, incorporating functional manifestations such as persistent lower-limb edema in addition to imaging findings, reflects real-world vascular practice and allows identification of clinically meaningful venous dysfunction that is frequently underrecognized. Venous-specific risk factors (e.g., obesity/BMI, liver disease, thrombophilia, prior VTE/PE, and prior major surgery) were not systematically available in this retrospective cohort and therefore could not be consistently included in baseline comparisons, which may introduce residual confounding. Detailed characterization of arterial disease severity using multimodal imaging strengthens the analysis of arterio-venous interactions in a cohort with severe limb-threatening ischemia.

Because hyperlipidemia was captured only when explicitly documented, its prevalence is likely underestimated in this high-risk population.

The single-center, retrospective nature of the study restricts causal interpretation and may limit the broader applicability of the findings. The high prevalence of venous disease should be interpreted in the context of a clinically oriented definition and a highly selected population with advanced PAD, and may not be applicable to patients with milder disease. Although venous duplex Doppler was not uniformly available, the inclusion of clinically documented edema was intentional and reflects functional venous dysfunction relevant to everyday practice. Lower-limb edema is a nonspecific clinical finding in patients with advanced PAD and multimorbidity, and residual confounding by cardiac, renal, nutritional, or inflammatory factors cannot be fully excluded despite careful clinical assessment. The observed association between concomitant venous disease and lower major amputation rates should be interpreted with caution. Key clinical variables related to infection severity, wound characteristics, timing of presentation, and revascularization intent were not systematically captured in this retrospective cohort and could not be adjusted for, limiting causal inference and mechanistic interpretation of this finding. A sensitivity analysis restricted to imaging-confirmed venous disease was not performed, as the reduced sample size would have substantially limited statistical power and interpretability. Future prospective studies with systematic venous imaging are warranted to further validate these findings. Limb-level analyses treated each lower limb as a separate analytical unit and did not formally adjust for within-patient clustering. As a result, independence of observations cannot be fully assumed, and the possibility of correlated limb-level outcomes within the same patient should be acknowledged. These analyses were therefore intended to be exploratory and descriptive, aimed at identifying limb-specific patterns and associations rather than providing definitive causal estimates.

The multivariable regression models should be interpreted with caution. The very high odds ratio and wide confidence intervals observed for inflow disease likely reflect sparse data in certain categories and collinearity among arterial severity variables in this cohort with advanced PAD. These findings underscore the exploratory nature of the regression analyses and the challenges of disentangling individual arterial predictors in end-stage disease.

### 4.2. Clinical Implications

The results of this study indicate that venous disease is common and clinically relevant in patients with advanced PAD and should not be overlooked during vascular assessment. Routine evaluation of venous pathology—particularly in patients with severe distal arterial disease—may improve understanding of limb pathophysiology and refine risk stratification beyond arterial anatomy alone. An integrated arterio-venous approach may further inform personalized management strategies, including wound care, edema control, and surveillance, with the potential to improve limb-related outcomes in patients with limb-threatening ischemia. Treatment strategies (endovascular, surgical, or conservative management) were recorded when applicable.

## 5. Conclusions

Concomitant venous disease is highly prevalent in patients with advanced peripheral arterial disease and is closely associated with markers of severe arterial involvement at both patient and limb levels. These findings support the concept that venous dysfunction represents an integral component of advanced limb-threatening ischemia rather than an isolated comorbidity. A clinically oriented assessment that includes functional venous manifestations may improve understanding of limb pathophysiology in advanced PAD and supports further investigation of integrated arterio-venous approaches in carefully selected patients, rather than a change in standard management strategies.

## Figures and Tables

**Table 1:** Baseline characteristics of the study population, stratified by venous disease presence.

Variable	Total (N = 241)	Venous Disease (+) (n = 166)	Venous Disease (−) (n = 75)	*p*-Value
**Age, years, median [IQR]**	71.0 [64.0–79.0]	70.0 [64.0–78.8]	73.0 [65.0–79.0]	0.47
**Male sex, n/N (%)**	163/241 (67.6%)	113/166 (68.1%)	50/75 (66.7%)	0.95
**Hypertension, n/N (%)**	115/122 (94.3%)	86/90 (95.6%)	29/32 (90.6%)	0.38
**Diabetes mellitus, n/N (%)**	121/128 (94.5%)	86/90 (95.6%)	35/38 (92.1%)	0.42
**Hyperlipidemia, n/N (%)**	16/25 (64.0%)	11/17 (64.7%)	5/8 (62.5%)	1.00
**Smoking history, n/N (%)**	38/47 (80.9%)	24/30 (80.0%)	14/17 (82.4%)	1.00
**Congestive heart failure, n/N (%)**	25/34 (73.5%)	17/22 (77.3%)	8/12 (66.7%)	0.69
**History of stroke, n/N (%)**	39/46 (84.8%)	27/31 (87.1%)	12/15 (80.0%)	0.67
**CKD on hemodialysis, n/N (%)**	25/35 (71.4%)	15/21 (71.4%)	10/14 (71.4%)	1.00

**Table 2:** Prevalence of venous disease.

Measure	n (%)
**Any venous disease (patient-level)**	166/241 (68.9%)
**Unilateral venous disease (patient-level)**	134/241 (55.6%)
**Bilateral venous disease (patient-level)**	32/241 (13.3%)
**Venous disease in the left limb (MS)**	118/241 (49.0%)
**Venous disease in the right limb (MD)**	50/241 (20.7%)

**Table 3:** Limb-level characteristics of peripheral arterial disease (PAD). (N = 482 limbs).

Limb-Level PAD Characteristic	Value
**Inflow disease, n/N (%)**	447/477 (93.7%)
**Femoropopliteal disease, n/N (%)**	313/477 (65.6%)
**Outflow disease, n/N (%)**	469/477 (98.3%)
**Multilevel disease, n/N (%)**	461/477 (96.6%)
**Segment occlusion score, median [IQR]**	4.0 [1.0–4.0]
**Tibial runoff count, median [IQR]**	1.0 [0.0–2.0]
**Patent pedal arch, n/N (%)**	155/461 (33.6%)

**Table 4:** Association between venous disease and PAD severity (limb-level analysis).

PAD Characteristic	Category	Venous Disease Present, n/N (%)	*p*-Value
**Multilevel disease**	Absent	2/15 (13.3%)	0.099
	Present	164/461 (35.6%)	
**Outflow disease**	Absent	1/8 (12.5%)	0.27
	Present	166/469 (35.4%)	
**Inflow disease**	Absent	5/30 (16.7%)	0.048
	Present	162/447 (36.2%)	
**Patent pedal arch**	Absent	125/306 (40.8%)	<0.001
	Present	38/155 (24.5%)	
**Segment occlusion score**	0–1	39/165 (23.6%)	<0.001
	2–3	8/30 (26.7%)	
	≥4	120/283 (42.4%)	
**Tibial runoff count**	0	112/188 (59.6%)	<0.001
	1	36/125 (28.8%)	
	2–3	19/164 (11.6%)	

**Table 5:** Treatment strategies and outcomes according to venous disease (patient-level).

Treatment/Outcome	Total n/N (%)	Venous Disease (+)	Venous Disease (−)	*p*-Value
**Endovascular therapy documented**	6/241 (2.5%)	4/166 (2.4%)	2/75 (2.7%)	1.00
**Thromboendarterectomy**	3/241 (1.2%)	2/166 (1.2%)	1/75 (1.3%)	1.00
**Angioplasty**	15/241 (6.2%)	13/166 (7.8%)	2/75 (2.7%)	0.16
**Any bypass surgery**	101/241 (41.9%)	75/166 (45.2%)	26/75 (34.7%)	0.16
**Amputation (any)**	70/241 (29.0%)	37/166 (22.3%)	33/75 (44.0%)	**0.001**
**Documented occlusion**	148/241 (61.4%)	102/166 (61.4%)	46/75 (61.3%)	1.00
**Death reported during evolution**	3/241 (1.2%)	1/166 (0.6%)	2/75 (2.7%)	0.23

**Table 6:** Multivariable logistic regression analysis for the presence of venous disease (patient-level).

Predictor	OR (95% CI)	*p*-Value
**Age (per 1 year)**	1.00 (0.97–1.03)	**0.92**
**Male sex**	0.90 (0.45–1.77)	**0.75**
**Max segment occlusion score (per 1 unit)**	0.12 (0.01–1.48)	**0.10**
**Min tibial runoff count (per 1 unit)**	0.09 (0.01–1.15)	**0.064**
**Any patent pedal arch**	3.00 (1.64–5.52)	**<0.001**
**Any inflow disease**	94.20 (2.55–3478.01)	**0.014**

## Data Availability

The data presented in this study are available on request from the corresponding author. The data are not publicly available due to patient confidentiality.
